# Knowledge diffusion within a large conservation organization and beyond

**DOI:** 10.1371/journal.pone.0193716

**Published:** 2018-03-01

**Authors:** Jonathan R. B. Fisher, Jensen Montambault, Kyle P. Burford, Trisha Gopalakrishna, Yuta J. Masuda, Sheila M. W. Reddy, Kaitlin Torphy, Andrea I. Salcedo

**Affiliations:** 1 The Nature Conservancy, Arlington, Virginia, United States of America; 2 College of Education, Michigan State University, East Lansing, Michigan, United States of America; 3 The Nature Conservancy, Quito, Ecuador; Rutgers The State University of New Jersey, UNITED STATES

## Abstract

The spread and uptake of new ideas (diffusion of innovations) is critical for organizations to adapt over time, but there is little evidence of how this happens within organizations and to their broader community. To address this, we analyzed how individuals accessed information about a recent science innovation at a large, international, biodiversity conservation non-profit–The Nature Conservancy–and then traced the flow of how this information was shared within the organization and externally, drawing on an exceptionally data-rich environment. We used surveys and tracking of individual internet activity to understand mechanisms for early-stage diffusion (knowledge seeking and sharing) following the integration of social science and evidence principles into the institutional planning framework: Conservation by Design (CbD 2.0). Communications sent to all employees effectively catalyzed 56.4% to exhibit knowledge seeking behavior, measured by individual downloads from and visits to a restricted-access site. Individuals who self-reported through a survey that they shared information about CbD 2.0 internally were more likely to have both received and sought out information about the framework. Such individuals tended to hold positions within a higher job grade, were more likely to train others on CbD as part of their job, and to enroll in other online professional development offerings. Communication strategies targeting external audiences did not appear to influence information seeking behavior. Staff who engaged in internal knowledge sharing and adopting “evidence” practices from CbD 2.0 were more likely to have shared the document externally. We found a negative correlation with external sharing behavior and in-person trainings. Our findings suggest repeated, direct email communications aimed at wide audiences can effectively promote diffusion of new ideas. We also found a wide range of employee characteristics and circumstances to be associated with knowledge diffusion behavior (at both an organizational and individual level).

## Introduction

### Background

Sustainability is a complex concept with multiple dimensions, and complex problems often require collaboration across stakeholders and sectors, seeking innovative approaches to both new and longstanding problems. The diffusion of innovations (the spread and uptake or adoption of new ideas, also referred to as “knowledge diffusion”) has long been credited for advances in conservation policy and practice [1,2]. The diffusion of innovation theory [3] is described in five stages: 1) knowledge (receiving information about new ideas, possibly seeking additional knowledge to gain understanding, but not necessarily being inspired to act on that knowledge), 2) persuasion (forming an opinion about the innovation, whether positive or negative), 3) decision (testing it out to decide whether or not to adopt it), 4) implementation (applying the innovation in one’s own context) and 5) confirmation (determining if the innovation is working, and either continuing to apply the innovation or ceasing to).

This process can occur both within the bounds of a formal system, such as an organization (internal or intra-organization diffusion) and between systems (external or inter-organization diffusion). Individuals and organizations facing sustainability challenges often rely on collaboration across sectors, partnerships, and coalitions. Within this context, individuals may choose to share knowledge about a given innovation (contributing to the awareness of others) after any of the five stages, whether or not they ultimately adopt and continue the practice. In our study, we focus on knowledge sharing (communicating with others about an innovation) and rather than the broader diffusion process (which goes beyond knowledge sharing and includes adoption or uptake).

Sharing knowledge in the pursuit of diffusing innovation can be a form of cooperation. Solutions to complex sustainability problems may not be obvious, and often require new innovations or evidence. Evidence based practices, embodying common language, and establishing a shared legitimacy are critical as organizations attempt to consistently address conservation challenges. Yet having the innovations spread and get adopted within organizations is paramount to their efficacy [4].

A large but disparate literature has examined the diffusion of innovation process and the stakeholders instrumental to the process. In particular, in the last two decades, research has examined predictors of who diffuses innovations, the role of social network factors in diffusing innovations, and intra- and inter-organizational models of diffusion. Research on the evolution of cooperation has found individual characteristics, such as reputation and behavioral diversity, are important predictors for promoting cooperation [5], and that people with high reputations (e.g., trusted as expert advisors) are crucial for that process [6]. Similarly, early empirical tests of the diffusion of innovations theory highlighted that individual characteristics (e.g., age, formal education) and behaviors (e.g., seeking written information, attending meetings and participating in other programs) could predict the likelihood an individual will engage in one or more stages of the diffusion process [2]. The Nature Conservancy (TNC), a large international biodiversity and environmental sustainability-oriented non-profit aims to influence practices and policies in arenas as diverse as sustainable agriculture and freshwater security. For such projects, evidence plays a critical role in demonstrating that conservation offers benefits to other stakeholders and partners, especially those for whom conservation may not be the primary goal (e.g., farmers, urban residents). This burden of proof is higher than working within the conservation sector because trust has to be built, and because the projects aim to deliver outcomes for biodiversity and people. It is thus critical to understand how new science ideas that build this evidence base are shared both within large conservation organizations and externally.

In other lines of research, studies have found social networks play an important role in promoting or hindering an individual from engaging in knowledge diffusion. For example, farmers in the so-called “corn belt” of the US Midwest are more likely to adopt conservation practices (e.g., cover crops and riparian buffers) if reinforced through local social networks and regional incentive programs [7]. Another study found that adopting forest conservation practices accelerated when landowners received information through different channels as they progressed through the stages of the diffusion process [8]. In sustainable water fund projects, water users and stakeholders (e.g., businesses, municipal governments, charitable organizations) fund watershed conservation projects. These individuals and organizations can come together around a common problem and adopt evidence based practices that work within their local context. Adapting the solution to their locality and building a coalition with sustained commitment helps these programs to attain their goals [9].

These findings reinforce research in other fields that shows that both closer proximity to the origin of information and similarity between the source and recipient of the innovation are important for knowledge diffusion [10,11]. However, there is less research on diffusion at large organizations, and the increasing reliance on online resources and availability of extensive digital datasets offers a new opportunity to address this research gap [12–14].

Several distinct responses can occur after an individual becomes aware of an innovation, including knowledge seeking (looking for information to learn more about an innovation), knowledge sharing (the spread of knowledge without a strong persuasive element), dissemination (planned efforts to convince a targeted group to adopt the innovation) and implementation (mainstreaming an innovation throughout an organization). These responses may all occur for some people, while others may only engage in some or none of them [15]. Individuals choose to share knowledge for a variety of reasons, and while much research asserts that these reasons relate to self-interest, such as being promoted at an organization by being seen as an expert [16], there may be other reasons associated with the expectations one has when sharing knowledge. Bock and Kim [13] found that employees in four large public organizations reported sharing more knowledge when they considered their efforts to positively impact the organization as a whole, or increase their relationships with others within their organization. Sharing knowledge *outside* of organizations may also improve relationships with people at other organizations, or be an opportunity to influence other organizations, and it may happen naturally during collaborations [17].

Despite the growing research on diffusion of innovations, there has been relatively little research examining inter-organizational diffusion, intra-organizational diffusion, and the factors that predict it within a single organization. This is important for several reasons. Decision-makers at organizations continuously strive for cost effective and efficient methods for creating dynamic organizations that respond to changing needs, and a study on a single organization can provide unique insights on the diffusion process. Further, organizations often seek to influence those around them, but how internal and external diffusion jointly occur is unclear.

To understand the dynamic process of information sharing within and outside an organization, we investigated how a new science-based innovation diffuses among employees in a large organization and from these employees to other organizations. We used the case of the evolution of the core science principles of TNC’s institutional planning framework (Conservation by Design 2.0, or CbD 2.0) to include social science and the use of evidence. In particular, we investigated the first two stages of the diffusion of innovation for inter- and intra-organizational diffusion by examining 1) which communication channels effectively improved awareness or knowledge of the innovation, and 2) the factors associated with individuals actively seeking more information about the innovation. A significant contribution of our study is the use of 16 datasets that capture multiple communication channels, individual characteristics of knowledge diffusers, and objective and subjective data on diffusion and adoption of the innovation (Table 1). Using these data, we identified characteristics of individuals who shared knowledge about the innovation, and explored the effect of different approaches to share knowledge about it. The combination of these datasets allowed us to provide internal validation. As we had limited time to directly observe diffusion, we completed this analysis with an investigation of how past versions of CbD diffused, drawing on a literature review and interviews.

**Table 1:** Summary of data sources evaluated for this study^a^.

Data Source	Data Type	Used	Notes
**Restricted-access Site (also referred to as "Connect") Visitors**	Individual	Yes	List of who accessed intranet pages related to CbD at what time (this site was only accessible by TNC staff)
**WebEx Attendance**	Individual	Yes	List of who attended the two CbD webinars, as well as who attended a webinar on human well-being (tracked separately)
**TNC Administrative**	Individual	Yes	Human resources data for all current employees, e.g. job grade, location, job family, sex, etc.
**TNC Training / Learning**	Individual	Yes	Which optional courses (whether online or in-person) were completed by each individual.
**TNC Labor (Billing Hours Worked)**	Individual	Yes	Used to determine which individuals worked on the same projects, which in turn was used to identify boundary spanners
**TNC Fundraising**	Individual	Yes	Total funds raised by each operating unit (OU: state or region) in North America, divided by staff in each OU. Control variable.
**Participation in review / drafting / piloting CbD documents & methods**	Individual	Yes	Used in calculating exposure to CbD 2.0. We gathered this email manually via email requests to the staff sending out requests for staff to provide various forms review, and distinguished between those who were invited and those who participated.
**Attendance of in-person CbD plenary or conference session**	Individual	Yes	Used in calculating exposure to CbD 2.0. We requested attendee lists from conference and session organizers to determine who learned about CbD 2.0 in person.
**Vertical Response**	Individual	Yes	Used in calculating exposure to CbD 2.0. This tool tracks which individuals clicked on newsletter stories, complementing the metric of who accessed intranet pages about CbD (which indicated that staff wanted to learn more after reading the newsletter story that this metric tracked)
**Survey**	Individual	Yes	Survey conducted via Qualtrics specifically to collect information about CbD 2.0
**Constant Contact**	Individual	Yes	Which individuals opened emails sent by executives, which included an invitation to the survey and a link to learn more about CbD. We also tracked who took the survey and clicked the link.
**Google Scholar / Web of Science**	Individual	Yes	Literature review of articles related to CbD (or citing it).
**Interviews**	Individual	Yes	Semi-structured interviews with key informants within TNC and other conservation organizations about diffusion of past versions of CbD.
**Conservation Gateway (Traffic)**	Aggregate	Yes	Page views and downloads by date on this public website of specific pages & documents related to CbD (primarily the full guidance document).
**Conservation Gateway (Visitors)**	Aggregate	Yes	Visitors by location, domain, state, city, only available for ~5% of traffic.
**Nature.org (Traffic)**	Aggregate	Yes	Page views and downloads by date on this public website of specific pages & documents related to an overview of CbD.
**TNC Email**	Individual	No	Would have allowed us to map social networks via anonymized email headers, and/or to identify knowledge sharing via anonymized keyword searches. Not used due to privacy concerns.
**TNC Expenses**	Individual	No	Expenses including equipment, travel costs, etc. Could have been used to identify patterns like which staff may have met in person (if they charged a travel expense in the same location on the same date), or used to bolster labor data about who worked on the same projects.
**CCNet Listserv**	Individual	No	Public listserv used by CCNet coaches; monitored with the expectation CbD 2.0 would be discussed there, but a past revision of the Open Standards had no discussion, and there was a single post about CbD 2.0 during our study period.
**Google Trends**	Aggregate	No	Intended to track interest in CbD over time—not used as data was unreliable (partly b/c there was insufficient traffic about CbD).
**PDF Tracking**	Aggregate	No	Would have allowed us to count # of times each pdf was opened to track sharing via email–not used due to legal and privacy concerns.
**Search terms**	Aggregate	No	# of searches each day on websites above on terms relating to CbD. Not used as it showed almost no variation over time.
**Bitly links**	Aggregate	No	Custom short trackable links to websites. Used at two in-person conferences to test whether any attendees would use the links to learn more about CbD 2.0, but there was almost no response.

^a^ Data sources either used in this study, or evaluated and rejected; individual data sources can be tied to a specific individual (which we always anonymized), aggregate data sources cannot.

To our knowledge this is the first study of its kind using such a rich and diverse suite of data to evaluate and build a more comprehensive picture of diffusion at a large organization. Organizational theory suggests that large, multi-national organizations tend to influence the diffusion of innovation process first within their sector (e.g. conservation or business), and then in other sectors [18], but we focus here on the conservation sector, as we believe our findings are likely to be most applicable to other conservation NGOs.

### Conservation by design: History and innovations

Since 1951, TNC has operated as a biodiversity-oriented conservation non-profit, which now has a presence in over 69 countries. One study found that the organization has the greatest assets and revenue among comparable organizations in the sector [19], and can be considered an influential actor. Some of the key science innovations at TNC are encapsulated in a document providing guiding principles and a planning methodology–Conservation by Design (CbD)–which is periodically updated to reflect advances in scientific understanding and its application to practice. After the first release in 1995, there have been ongoing efforts to both disseminate and implement the science ideas and practices via hiring dedicated staff [20], creating a network of volunteer “coaches” to conduct internal trainings, and formalizing a network with external organizations to promote diffusion and learning exchange (Conservation Measures Partnership, later the Conservation Coaches’ Network aka CCNet) to mainstream these concepts across the sector [21]. In 2004, many of the core elements of CbD were incorporated into the new Open Standards for the Practice of Conservation[22], which in turn has been adopted by several organizations [23] and continues to evolve over time. There is also a substantial body of peer-reviewed literature about CbD that we analyzed to gauge interest in prior versions outside of TNC.

The most recent revision to the document (CbD 2.0) was released in March 2015 (with more detailed technical guidance released in March 2016). The revisions focused on updating the conservation planning process and integrated social science and human well-being objectives, outlined a method for robust evidence assessment, provided guidance for targeting systemic drivers of ecological stress rather than local symptoms, and highlighted the importance of spatially explicit identification of conservation strategies [24]. This was considered a major revision; each of these elements has not been systematically applied, and CbD 2.0 codified these elements as universal and substantially more robust. For example, while it has been fairly common at TNC to develop a theory of change, assessing the evidence-base for each step in a theory of change is novel.

## Methods

This study was approved by Michigan State University’s Human Research Protection Program (Institutional Review Board). The program can be reached at 517-355-2180 or irb@msu.edu, and the chair is Harry McGee, M.P.H. Data requests should be submitted to irb@msu.edu. Although not required, it was also reviewed by The Nature Conservancy's Ethics and Compliance department, as well as the Office of the Chief Scientist. Written consent was obtained as applicable. Questions for The Nature Conservancy about data or privacy protections can be sent to Josh Goldstein at science@tnc.org.

We have broken the methods section into several sections, all focused primarily on behavior related to sharing knowledge about CbD 2.0 and seeking knowledge about CbD 2.0. First, we conducted an analysis of diffusion of past versions of CbD to other organizations, using both semi-structured interviews with individuals playing key roles in prior versions of CbD, as well as a literature review of papers published about prior versions about CbD. We examine internal and external diffusion separately, although the methods are largely similar. In both cases, we first assessed the extent of knowledge seeking behavior for CbD 2.0 using data from email tracking and website metrics. We then investigated the characteristics of staff who diffuse the innovation both within and outside of TNC by estimating a logistic regression where we regress self-reported behavior of internally sharing information about CbD 2.0 on individual characteristics of TNC staff. In the following sections, we expand on the data and methods for each set of analyses.

### Diffusion of prior versions of CbD

To better understand how past versions of CbD have spread to other organizations, we conducted an analysis examining both the peer-reviewed literature citing different versions of CbD, and interviewing staff affiliated with previous revisions both from TNC and partner organizations. This analysis is intended to complement the study of CbD 2.0 (given that we only had a short period of time to observe diffusion specifically for CbD 2.0) by examining later stages of diffusion for prior versions of CbD.

Reviewing citations of past versions of CbD was intended to understand both how widely formal knowledge sharing occurred through this communication channel over time and the extent to which actors across sectors (e.g. academic, government, other NGOs) may have been involved. We considered citations in both the academic citation search engine Google Scholar (GS), which searches through the full text of documents, and the academic citation database Web of Science (WoS), which is indexed according to predefined fields and journals. We anticipated the CbD was more likely to be discussed or cited within the text of the document and thus chose GS as the primary search platform. Comparisons between the two citation search platforms suggests that in many cases at least one novel and important citation was found through WoS that was not found in the same GS search, so we used WoS as a supplementary search. Our GS search entered, "Conservation by Design" AND "Nature Conservancy," in the “exact phrase” field. The radio box “anywhere in the article” was selected; dates were constrained to 1995–2015. We found 288 citations. We then conducted the same search in WoS and combined the novel results for a total of 295 citations. We then eliminated obvious duplicates and citations in languages other than English resulting in 175 references. We then searched for the full text version of each document, eliminating 9 documents that could not be found online. We downloaded the remaining 164 and read each article and eliminated those that did neither cite CbD in the bibliography nor discuss the document in text, figures and tables and were left with a total of 151 publications that cite and/or discuss CbD (See S1 File for the complete list). Within these publications, we examined the diversity of institutions that authored articles citing CbD, hypothesizing that most papers would be authored by TNC staff, followed by academics, followed by other NGOs.

We also conducted semi-structured interviews with eight key informants both within TNC and the broader conservation sector. These individuals were selected for their authorship/leadership of previous versions of CbD and/or their participation in processes that led to the diffusion of these ideas to other institutions (e.g., CMP, CCNet). While this is quite a small sample size, they collectively had over 260 years of experience in conservation, and we included all the staff who played the most critical roles related to CbD within the organization.

The focus of the interview questions was to better identify prior methods for knowledge-sharing, dissemination, and implementation of CbD that were applied in the past two decades. We asked respondents three questions: 1) “how did past versions of CbD spread internally and externally, and what key factors either aided or hindered that process?”, 2) “How do you think CbD 2.0 fits into CCNet and the open standards, and do innovations primarily still come from TNC, or primarily from other organizations?”, and 3) “What has made past innovations not only get adopted but persist over the long term (internally and externally)?” Our purpose with these interviews was to elicit anecdotal evidence of the extent to which these methods led individuals and organizations to a decision to adopt past innovations, as this stage of diffusion is notoriously difficult to assess empirically [3].

While this analysis allowed us to look at past diffusion, the majority of our study focused on the initial phases of internal and external diffusion of CbD during the drafting phase and shortly after its release. The full technical guidance of CbD 2.0 was not released publicly until March 2016 (allowing for slightly over two months to observe external diffusion as we collected data through May 31, 2016), although earlier drafts were shared with some staff starting in October 2015, and a high-level summary of the revisions was publicly released in March 2015 (providing an opportunity for us to observe early external diffusion).

### Internal knowledge sharing and knowledge seeking

We examined two issues around internal knowledge: which forms of communication led to the most internal awareness and knowledge seeking about CbD 2.0, and the characteristics of staff who shared information internally. The first set of analyses focus on administrative and secondary data sources to directly observe whether staff sought out more information after events of drawing attention to CbD 2.0, such as organizational newsletters and presentations at external conferences. The second set of analyses use primary data from a survey of all conservation, science, and executive staff within the North America program of TNC (1,536 staff) conducted via Qualtrics in May 2016 (the survey instrument is available in S2 File).

The survey asked questions about conservation practices, informal networks, and about how respondents received and shared information about CbD 2.0 with their intra-organizational colleagues and with external individuals across conservation and other sectors (note that this survey required written consent to participate in this research). This survey is a form of indirect data as responses were declared rather than empirically observed. Prior to testing, the survey was validated with individuals who were ineligible to be surveyed (n = 16). This included people that worked on the CbD 2.0 guidance (n = 9) and could provide information on whether the survey captured new practices/innovations, as well as people outside the study area (n = 7). Their feedback was used to refine and clarify the survey questions.

The response rate was 44.7% percent (n = 686 completed the survey). The final sample was not representative of the North America program (S1 Table). Individuals in this sample had a higher job grade, were more likely to work for a regional program, and less likely to be in the conservation job family as compared to the population of staff in the North America program.

### Extent of internal knowledge seeking

We investigated the extent of internal knowledge seeking by examining a variety of online tracking data between January 1, 2015 and May 31, 2016. By observing three different pathways of diffusion, we assessed the attention and exposure to CbD 2.0 following events drawing attention to it. We first assessed the changes (or lack of change) in unique daily visitors to a TNC intranet website about CbD 2.0 (only accessible by TNC staff) on dates when events related to CbD 2.0 took place (indicating that they are seeking additional knowledge). Next, we measured the number of people who attended two online webinars about CbD 2.0 that was advertised to all TNC staff. The first webinar was held in March 2015 (at three different time slots over two days), and the second was held in April 2016 (also at three times over two days). Webinar attendance was voluntary, so we believe this measure captures changes in interest in CbD 2.0 over one year. Finally, we tracked the number of TNC staff who opened at least one of four emails sent out by three different senior executives of TNC containing a link to information about CbD 2.0 (as part of an invitation to take a survey related to this research). This both assessed how large a subset of our target population had an opportunity to read the email and consider taking the survey, and investigated the effectiveness of emails sent by TNC executives as a way to reach large numbers of staff.

### Characteristics of internal knowledge sharing

We estimated a logistic regression where we regress self-reported behavior of internally sharing information about CbD 2.0 on individual variables associated with characteristics of TNC staff. The regression can be represented as:

$$\ln\frac{p}{1-p}=\beta_{0}+\sum_{i=1}^{n}\beta_{i}*x_{i}$$

where *p* is the probability of internal knowledge sharing, β_0_ is the intercept, and β_i_ is the regression coefficient for predictor variable *x*_*i*_. See Table 2 for the full list of variables used.

**Table 2:** Descriptive statistics of the variables evaluated in model of characteristics associated with internal diffusion. See S2 Table for more detail about each variable.

Variable	Mean	SD	Min	Max
***Dependent variables***				
Individual shared info internally (%)^a^	24.3	42.9		
Individual shared info externally (%)	35.4	47.8		
***Independent variables***				
*Characteristic*: *Sharing information*				
Shared science/technical information internally (%)	86.4	34.2		
Shared science/technical information externally (%)	82.4	38.0		
*Characteristic*: *Formal role/job in knowledge sharing*				
Is a CCNet coach (%)	6.12	24.0		
*Characteristic*: *Awareness*, *knowledge and exposure*				
# of online trainings completed	6.29	6.01	0.0	54.0
# of in person trainings completed	0.519	1.84	0.0	14.0
CbD awareness (passive)^b^	2.13	0.707	1.0	5.0
CbD knowledge seeking (active)^b^	1.73	1.33	0.0	8.0
*Characteristic*: *Strong social network*				
Is a boundary spanner (%)	17.5	38.0		
Years working in conservation	16.5	10.2	0.0	44.0
# of internal collaborators	8.56	4.26	0.0	15.0
Service years at TNC	10.6	8.22	0.175	36.6
*Characteristic*: *Changes in practice post CbD 2*.*0*				
Incorporates evidence due to CbD 2.0 (%)	12.9	33.6		
Incorporates uncertainty due to CbD 2.0 (%)	14.8	35.6		
*Characteristic*: *Existing alignment/identification with innovation*				
Prior “people” practices^b^	4.33	2.35	0.0	8.0
Prior “evidence” practices^b^	3.73	2.41	0.0	9.0
Prior “systematic change” practices^b^	1.43	1.06	0.0	3.0
*Characteristic*: *Communication*				
Percent of time spent in communication	18.2	17.9	0.0	100
# of staff communicated with	9.08	3.73	4.0	15.0
Control variables				
Gender^c^^d^				
Male	*385*			
Female	*300*			
Job grade (higher is more senior)	6.63	2.17	1.0	13.0
Years post-secondary education	5.79	0.893	2.0	8.0
Operating Unit (OU) size (# of staff)	68.1	80.7	9.0	1087
Budget per person (USD)	$127,612	$95,147	$1,146	$597,344
OU Type^d^				
Worldwide office	3			
Regional	70			
State	*612*			

The seven hypothesized characteristics are identified in the table with italic text, with the specific variables associated with each characteristic listed underneath.

^a^ All binary variables are indicated with (%) after the variable name, and not including minimum and maximum values.

^b^ This is a sum of several different binary variables that each relate to this overall topic; all available variables expected to have a strong relationship to the characteristic were utilized, see S2 Table for details on each variable.

^c^ All respondents identified as either male or female, no additional options were presented.

^d^ Treated as a categorical variable, with number of staff in each category listed below.

Based on a literature review, we initially hypothesized seven main characteristics that describe individuals who are more likely to share knowledge (listed below). We then evaluated all of the specific variables we had data for, identifying the subset of them that appeared to be good proxies of these characteristics and tying each selected variable to the related characteristic. These hypothesized characteristics and associated observed characteristic variables were used to develop statistical models to test the degree to which they were associated with internal and external knowledge sharing. Respondents were directly asked whether or not they shared information about CbD 2.0 internally and externally, providing us dependent variables about knowledge sharing to compare to these characteristics.

The first characteristic is having recently shared science and technical information with others, which we represented with survey respondents reporting that they had shared conservation science information internally and/or externally. Second is individuals with a formal job role that includes sharing knowledge [25], our chosen variable (CCNet coaches) specifically indicates a job role to promote CbD. Third, we considered general knowledge seeking behavior (such as optional trainings, [26] and increasing awareness of the innovation (through both passively receiving information and actively seeking it out). Note that although awareness on its own is insufficient to drive diffusion [27], we reasoned that one cannot share knowledge one has not received, and utilized variables related to both participating in trainings (online or in person), and receiving and seeking knowledge specifically about CbD 2.0. The fourth characteristic was people with strong social networks, primarily “boundary spanners” connected across different groups that are otherwise not well linked as they have been found to be more likely to share knowledge [28,29]. Given the complexity of conducting social network analysis and identifying clusters and boundary spanners, two other manuscripts have been submitted for publication describing how we accomplished that for this project. Other variables presumed to relate to strong social networks were years working in conservation (at TNC and overall) and the number of internal collaborators reported. Diffusion theory states knowledge sharing about an innovation is more likely when an innovation has been tried and been successful [11]. For this reason, we included a fifth characteristic for having adopted CbD 2.0 practices after reading it, using two variables that indicated users had changed their practices as a response to reading CbD 2.0. Sixth, we included the characteristic of already using practices that align with the new innovation (as they may be more prone to pass on information that validates their existing beliefs, [30]), using three variables that counted how many specific practices aligned with CbD 2.0 they were already using. Finally, we included a characteristic for staff who reported spending more time communicating with others about any topic (and/or communicating with more people, reported as two separate variables [31]). We also included control variables including gender, job grade, education, and details about their operating unit / department (type, size, and budget). See Table 2 for a list of all considered characteristic variables (grouped by the seven hypothesized characteristics above) with descriptive statistics, and S2 Table for more details on variable definitions and descriptions and how they were compiled. We have used short variable names here to be concise, e.g. “CbD awareness (passive)”.

We estimated the importance of these observed characteristic variables on the binary dependent variable “Individual shared info internally” (Table 2) taken from the survey. Using a logistic regression modeling approach, we calculated the odds of sharing knowledge internally for each characteristic variable (the odds defined as the ratio of the probability of an individual sharing information internally to the probability of an individual not sharing knowledge internally).

### External knowledge sharing and knowledge seeking

To investigate external knowledge sharing (knowledge about CbD being shared by individuals at TNC to others outside of TNC), we took a similar approach to internal knowledge sharing.

### External dissemination and knowledge seeking

Data for this analysis come from two publicly accessible websites that have information about CbD 2.0: nature.org and the Conservation Gateway. Nature.org is TNC's primary public website, and an overview of CbD 2.0 was published there in March 2015. Conservation Gateway is a public TNC website for conservation practitioners, and it houses older versions of CbD and is where the CbD 2.0 guidance was published in March 2016. Using data from these websites, we created a time series of page views and downloads of the PDF CbD guidance documents for the pages relating to CbD on each site. If external diffusion was widespread, we would expect to see higher traffic on these public pages than on internal TNC pages given that the potential external audience is much larger. For example, if a given internal page was viewed 50 times by TNC staff, and an equivalent public page was viewed 5,000 times, that could show broader interest from more people outside of TNC (even if a higher percentage of TNC staff are interested, higher absolute views for a public page indicates external knowledge seeking). Again, we assessed the change in web traffic with relation to CbD events as a potential indicator of external interest, recognizing that some of this traffic likely is by TNC staff. Lastly, we compiled data on all traffic to the Conservation Gateway that came from different countries and focused on countries where TNC does not work to get an estimate of traffic we can be fairly confident came from non-TNC individuals.

### Characteristics of external knowledge sharing

As with internal knowledge sharing, we used a logistic regression modeling approach to characterize those who shared knowledge about CbD 2.0 externally (the same equation applies). Again, we determined the odds of sharing knowledge externally for each observed characteristic variable as the ratio of the probability of an individual sharing information to the probability of an individual not sharing knowledge externally. While we used all the observed characteristic variables included in Table 2 as covariates (including the same grouping into seven characteristics), there were some minor differences. Having shared CbD 2.0 knowledge internally is an additional covariate (under the “sharing information” characteristic), and the new dependent variable is whether CbD 2.0 knowledge was shared externally (given a value = 1) with 35.3% of the survey recipients indicating that they had shared CbD 2.0 material externally and approximately 4.37% not answering this question (Table 2). All statistical analysis was completed in the statistical environment R v.3.3.0 [32].

## Results

### Diffusion of prior versions of CbD

**Fig 1:**
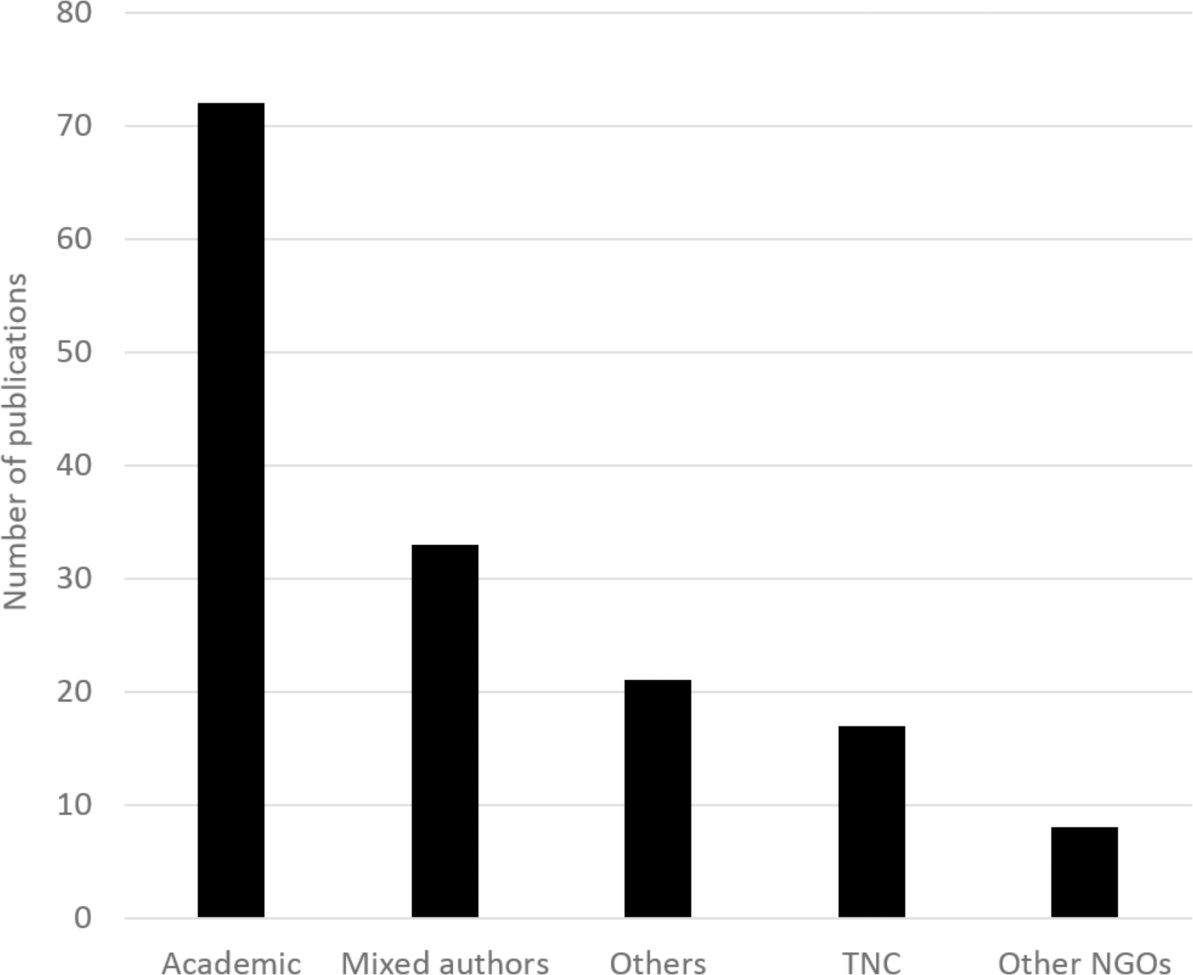
Total publications mentioning CbD. Publications are grouped by organization type of the authors. Except for “Mixed authors” all categories indicate that all authors on each paper were from the same type of organization (academic, TNC, NGOs other than TNC, or a different kind of organization).

Of the 151 articles that mentioned CbD: 16.6% (n = 25) had CbD as the primary topic of the paper, 53.0% (n = 80) discussed CbD in the text, and 30.5% (n = 46) cited CbD in the bibliography without discussion. 72 articles were written exclusively by people in academic institutions, 21 were authored solely by individuals at a government agency or independent research institute, 17 were written by only TNC staff, 8 were authored solely by employees of other NGO's, and 33 included authors from different types of institutions (Fig 1). These results suggest that in the past CbD innovations have diffused both outside of TNC and across different sectors. Based on the time-lag of citing the original version CbD, it is far too early to observe citations of CbD 2.0, however, we might predict similar patterns over time with CbD 2.0.

While semi-structured interviews included discussion of all the phases of diffusion, most interviewees focused specifically on adoption and implementation. Over half of the interview respondents listed the same six specific factors as important to promote diffusion (including adoption): bringing in partners early to develop and test methods, committing up front to sustain support for the planning methods, having in-person workshops, using peer-review and shared learning, providing financial support, explaining how the methods address *existing* needs planners already have, and the existence of CCNet as a support and learning network. These findings complement a previous informal survey of 73 participants in CCNet who suggested detailed written guidance, downloadable case studies, online trainings and webinars were helpful forms of support [33].

Several other factors were identified by interviewees as having promoted the adoption of new methods and tools in past iterations of CbD; these factors can be grouped into five categories. 1) Indirect support (developing written guidance documents, tools and information systems, templates, online self-guided training, etc.), 2) direct support (in-person training workshops with funding available to cover costs of attendees, phone / email support, peer review, etc.), 3) encouragement via professional networks (whether a mandate from executive leaders or peer pressure from colleagues in similar roles, as per [34–37], 4) partner inclusion (inviting partners to participate in developing and testing the methods, getting their feedback and ideas, setting a collaborative tone rather than assuming we have all the answers), and 5) persistence (sustaining the efforts above over time, scheduling follow-ups after each training and workshop to report on progress, refine efforts based on feedback from participants, etc.).

All eight interviewees indicated a strong belief that knowledge sharing alone (sharing a document without incorporating the factors mentioned above to promote diffusion) would not be successful in promoting internal or external diffusion, and five of them independently mentioned the same example of one specific innovation at TNC that was not widely adopted because of a lack of several of the factors above. Some other factors mentioned hindering diffusion included changing the language used to refer to the same (or similar) terms, criticizing earlier versions of the methodology. One person mentioned that TNC has nearly doubled in size from 1995, which could make internal diffusion more challenging.

Five interviewees mentioned that they expected external diffusion of CbD 2.0 so far to be minimal, primarily because TNC has not provided much guidance or actively promoted it and therefore external audiences would not understand how these new methods would or should be used.

### Internal knowledge sharing and knowledge seeking

#### Extent of internal knowledge seeking

Multiple measures suggest there was substantial interest and exposure to CbD 2.0; all staff received some information by email (whether or not they read it), 3,095 individuals (78.7% of all TNC staff) sought knowledge about it (e.g. opening an email, reading a newsletter, attending a webinar, etc.), and 1,984 individuals (50.5% of all TNC staff) specifically visited the intranet site with information about CbD 2.0 during the study period. Staff who did *not* seek knowledge about CbD 2.0 were more likely to work for an international operating unit (Africa, Latin America, or Asia-Pacific), to have worked for TNC for less time (a mean of 4.61 years compared to 8.02 years for all staff), and to work for human resources. Staff in the science or executive job families were the most likely to seek knowledge about CbD 2.0, as were individuals who worked for the North America region (where communication efforts were focused).

**Fig 2:**
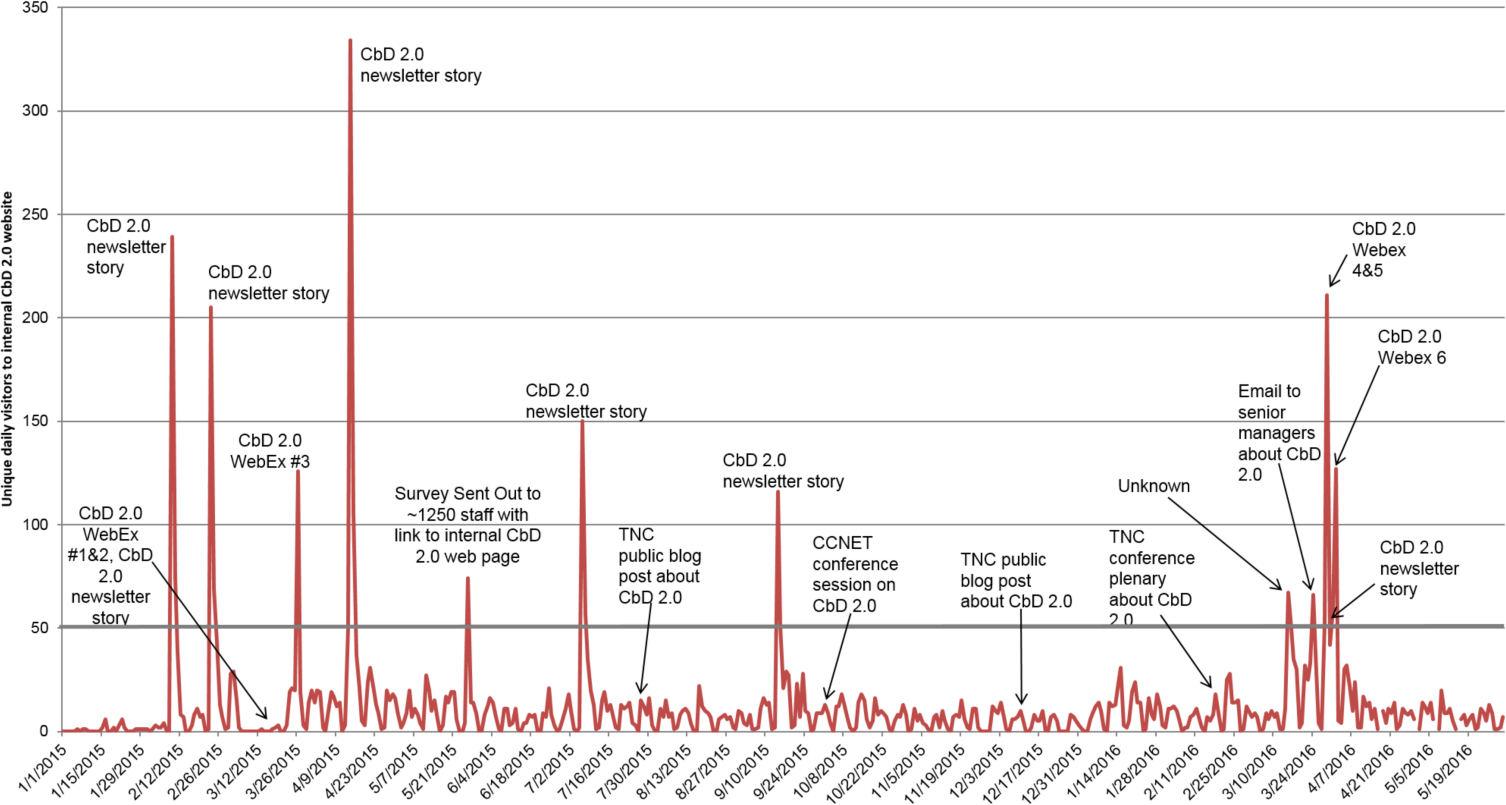
Unique daily visitors on internal website about CbD 2.0 (only accessible by TNC staff). Related events are noted via text labels, and the threshold of 50 daily visitors is shown in green.

There were 17 events (e.g. webinars, mass emails being sent, conferences, etc.) where the organization drew special attention to CbD 2.0 during the study period, and we observed substantial increases in traffic (defined here as over 50 unique visitors per day, which was outside of the range of normal traffic, which had a mean of 12 visitors per day) to the internal website following 12 of these events (Fig 2); no substantial response was observed to the other 5 events.

Of the 12 events with substantial increases, six occurred at the same time as organization-wide newsletters with stories about CbD 2.0, and three of them were from online webinars (WebEx sessions) about CbD 2.0 which all staff were invited to. Of the remainder, one was a baseline survey sent out as part of this research project in March 2015 (which included a link to the online web page and was sent to about 1,250 staff), one was an email sent from a TNC executive to several other executives (which was widely forwarded; it did not link to the internal website directly but nonetheless led to traffic there), and one did not correlate with any event we are aware of. These data suggest events that events which drew considerable attention from staff all targeted large numbers of staff, made it easy to learn more (providing a direct link), and had low time or opportunity cost. Another commonality is that interest waned quickly after each event rather than slowly tailing off, although overall interest and response to events did not decrease throughout the study period.

Note that due to a failure in site tracking in mid-March, we did not observe a response to two simultaneous events (a newsletter story and WebEx session in March 2015) that other data tracking sources indicated were heavily viewed. Also, no change in web traffic was seen in response to two public blog posts (on TNC's Cool Green Science blog), nor from two conferences (one was mostly non-TNC and had a small optional session about CbD 2.0, the other was TNC only and all ~400 attendees attended a plenary about CbD 2.0, which had a link to the intranet page). This lack of change indicates that in-person events may lead to less follow-up learning than simpler outreach such as newsletters and webinars; this may also be because newsletters and webinars are both received online and thus allow for immediate follow-up if interested, while in-person events allow less opportunity to quickly learn more online.

With respect to the online webinar series, 39.6% of all TNC staff around the world attended the first webinar (which was offered at 3 different time slots over two days). The attendance increased to 50.0% in the second online webinar (also offered at 3 time slots). As noted above, these sessions led to increased traffic on the CbD 2.0 website, and were also the only events that led to an increase in searches within the internal website for "conservation by design" and related terms. As TNC is a relatively decentralized global organization with almost 4,000 staff across 69 countries, 50.0% attendance is unusually high. By comparison, high profile events to which all staff are invited on average garner 16.5% attendance, suggesting that CbD is a topic of interest to TNC staff.

As noted above, emails sent from executives are often believed to be very widely read. Of the 1,536 staff who received a series of four emails inviting them to take our 2016 survey (an invite and three reminders, from three different executives), 51.2% opened at least one of the four emails.

#### Characteristics of internal knowledge sharing

Overall, 212 individuals (30.9%) of survey respondents were identified as internal knowledge-sharers. Results from the logistic regression model indicates the odds of sharing knowledge were greater for individuals who are CCNet coaches (this variable had the largest coefficient of 0.881, Table 3). As expected, the odds of sharing knowledge internally were higher for staff who sought knowledge about CbD (and presumably were interested in the topic) and those who were aware of CbD 2.0 principles because they had received information about it. Individuals whose prior conservation practices related to including people in conservation (aligning with CbD 2.0 principles) had greater odds of sharing knowledge internally. Job grade was also significant; each unit increase in job grade led to increased odds sharing information internally. The last positive factor was that for every additional online training module that an individual participated in, their odds of sharing knowledge internally increased. Conversely, staff with more years of experience doing conservation work had lower odds of sharing knowledge internally.

**Table 3:** Logistic regression results for internal knowledge sharing ^a^.

Variable	Coefficient	Standard Error	Odds Ratio
(Intercept)	-8.276***	1.15	0.000
Shared science/technical information internally	0.579	0.376	1.79
Is a CCNet coach	0.881*	0.377	2.41
# of online trainings completed	0.0347*†*	0.0181	1.04
# of in person trainings completed	-0.0895	0.0590	0.914
CbD awareness (passive)	0.414*	0.170	1.51
CbD knowledge seeking (active)	0.291**	0.0890	1.34
Is a boundary spanner	0.201	0.261	1.22
Years working in conservation	-0.0451**	0.0169	0.956
# of internal collaborators	0.0533	0.0465	1.06
Service years at TNC	0.0312	0.0193	1.03
Incorporates evidence due to CbD 2.0	0.104	0.382	1.11
Incorporates uncertainty due to CbD 2.0	0.286	0.339	1.33
Prior “people” practices	0.131*	0.0632	1.14
Prior “evidence” practices	0.0103	0.0596	1.01
Prior “systematic change” practices	0.0477	0.125	1.05
Percent of time spent in communication	-0.00701	0.00694	0.993
# of staff communicated with	0.00241	0.0431	1.00
Being a male (gender)	-0.246	0.225	0.781
Job grade	0.456*	0.0792	1.58
Years post-secondary education	0.138	0.146	1.15
Operating Unit (OU) size (# of staff)	0.00134	0.00241	1.00
Budget per person (USD)	-9.98E-07	1.11E-06	1.00
State OU (as opposed to Regional OU)	0.119	0.359	1.13
Worldwide Office OU (as opposed to Regional OU)	-3.895	3.10	0.020

^a^ Significant variables are identified with the following footnotes. See Table 2 for the characteristics associated with each variable.

*** p<0.001.

** p<0.01.

* p< 0.05.

† p< 0.1.

### External knowledge sharing and knowledge seeking

#### External dissemination and knowledge seeking

**Fig 3:**
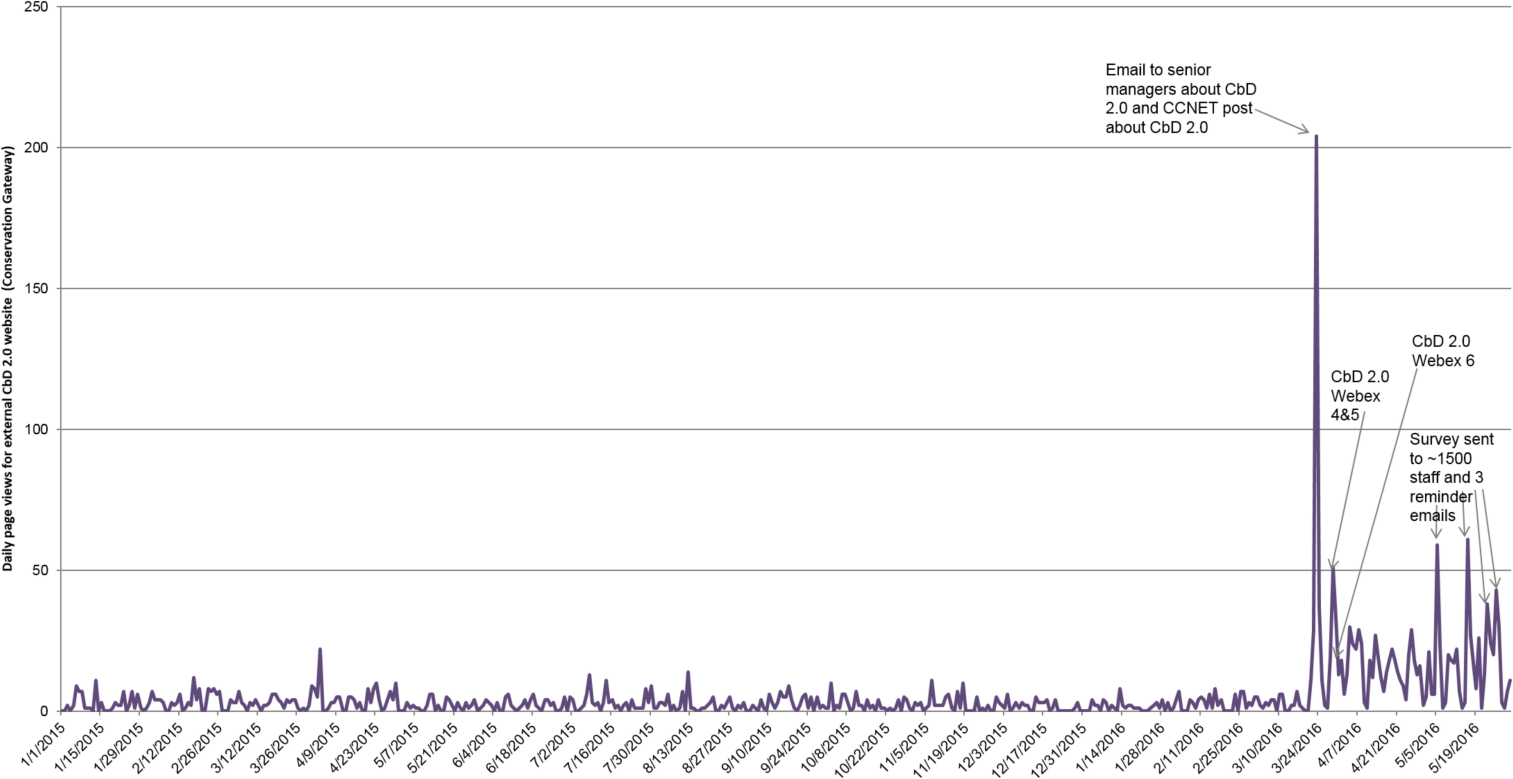
Daily page views for Conservation Gateway web pages about CbD 2.0. Related events are noted via text labels.

For the public-facing websites (accessible by anyone including non-TNC staff), we found it impossible to clearly differentiate between TNC staff traffic and non-TNC staff traffic. Fig 3 shows daily traffic to CbD-related pages on the Conservation Gateway over time; prior to March 23, 2016, this consisted of three separate web pages with information about past versions of CbD. On March 23, the full CbD 2.0 guidance was posted exclusively to the Gateway (not to any other websites). That day (which had the most traffic on a single day during our study period), there was both an internal event and an external event that together likely drove the increase. An executive at TNC sent emails directly to several other TNC executives with a link to the guidance document (which were widely forwarded throughout TNC, as some staff received it after it had been forwarded multiple times), but on the same day a link to the guidance document was posted to a public CCNet listserv. Accordingly, while we see increased traffic in response to internal events (such as WebEx meetings and the survey invite), we cannot attribute any substantially increased traffic definitively to external sources. While traffic was consistently low prior to March 23, 2016 (and was higher afterwards even in-between major events), the analytics tool used to collect this data was updated in early March 2016, and technical staff indicated prior data was less reliable.

While only 5% of web traffic on the Conservation Gateway had metadata indicating country of origin or domain (43 of 846 users), we did find some evidence of external diffusion. For the 43 visitors we could track, 13 of them (30%) were from countries where TNC does not have any staff located; some of the remaining 30 could well be non-TNC staff as well, but we had no way to verify this.

**Fig 4:**
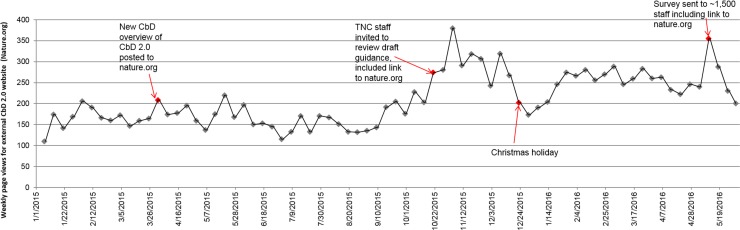
Weekly page views for nature.org external web page about CbD 2.0. Related events are noted via text labels.

For roughly the first half of the study period, traffic to the web page on nature.org (the other public website) with information about CbD 2.0 consistently varied between roughly 100–200 page views per week (Fig 4). Posting an overview document about the CbD 2.0 revision in March 2015 did not lead to a substantial increase in traffic, indicating either an initial lack of interest (perhaps because general members may find planning methods uninteresting) or the need for communications to draw attention to it (which were absent at that time). The first substantial increase came when TNC staff were invited to review a draft version of the CbD 2.0 guidance; the guidance document was not publicly available but did link back to the overview on nature.org. This increased traffic was sustained over several weeks, roughly the same period during which staff were working on submitting comments. *After* this period, the baseline traffic was consistently higher (250 to 300 page views weekly), some of which could have been due to external diffusion, but there were no spikes correlated with external events. Finally, our 2016 survey invitation, which included a link to the nature.org page, also appears to have spurred increased traffic.

#### Characteristics of external knowledge sharing

230 individuals (33.5% of the sample) were identified as external knowledge-sharers from the 2016 survey responses, of which 118 also shared knowledge internally. Five significant characteristic variables associated with this behavior were found in the logistic regression model (Table 4), four of which had a positive effect and one of which had a negative effect.

**Table 4:** Summary of logistic regression model characterizing external knowledge sharing^a^.

Variable	Coefficient	Standard Error	Odds Ratio
(Intercept)	-3.343***	0.873	0.035
Shared knowledge about CbD 2.0 internally^b^	0.883***	0.226	2.42
Shared science/technical information externally	0.151	0.289	1.16
Is a CCNet coach	0.213	0.386	1.24
# of in person trainings completed	-0.159**	0.0587	0.853
# of online trainings completed	0.0251	0.0167	1.03
CbD awareness (passive)	-0.00455	0.146	0.996
CbD knowledge seeking (active)	0.0567	0.0810	1.06
Is a boundary spanner	0.0759	0.245	1.08
Years working in conservation	0.00193	0.0128	1.00
Service years at TNC	0.0198	0.0161	1.01
Incorporates evidence due to CbD 2.0	0.826**	0.320	2.29
Incorporates uncertainty due to CbD 2.0	0.283	0.298	1.33
Prior “people” practices	0.0989*†*	0.0538	1.10
Prior “evidence” practices	0.153**	0.0528	1.17
Prior “systematic change” practices	0.0501	0.109	1.05
Percent of time spent in communication	0.000685	0.00565	1.00
Being a male (gender)	0.167	0.199	1.18
Job grade	0.0150	0.0649	1.02
Years post-secondary education	0.121	0.123	1.13
Operating Unit (OU) size (# of staff)	0.00266	0.00212	1.00
Budget per person (USD)	0.000	0.000	1.00
State OU (as opposed to Regional OU)	-0.517	0.332	0.596
Worldwide Office OU (as opposed to Regional OU)	-1.972	2.61	0.139

^a^ Significant variables are identified with the following footnotes. See Table 2 for the characteristics associated with each variable.

*** p<0.001.

** p<0.01.

* p< 0.05.

† p< 0.1.

^b^ Note that we used this dependent variable for internal knowledge sharing as an independent variable for the external knowledge sharing model.

First, the odds of sharing knowledge externally were greater for all staff who also shared CbD 2.0 information internally. We also found that evidence based practices (both prior to CbD 2.0, and adopted after reading CbD 2.0) increased external sharing. Also, individuals whose prior conservation practices related to including people in conservation (aligning with CbD 2.0 principles) had greater odds of sharing knowledge externally. Conversely, for every additional in-person training an individual participates in, the odds of sharing knowledge externally decreased.

## Discussion

Collectively, our findings revealed several insights about the processes of knowledge seeking and sharing, both internally and externally. While much of this study is focused on the early phases of diffusion of CbD 2.0, the analysis of diffusion of prior versions of CbD provides some insight as to what might happen next. Our analysis of scholarly publications revealed that past versions of CbD have been externally shared via peer-reviewed publications. Most papers were written without TNC staff involvement and demonstrates that external knowledge sharing of CbD has taken place, and that there is enough external interest in CbD to drive publications. This finding is especially noteworthy as CbD is primarily used by TNC, with most other organizations choosing instead to adopt the Open Standards for the Practice of Conservation (the modified version of CbD that removed TNC-specific language and concepts). Although we did not quantify the spread of the Open Standards through peer-reviewed literature, it is arguably another way that CbD has spread externally.

The interviews identified several factors that can either aid or hinder the process of knowledge diffusion. Several of these ingredients were also found to be significant in the analysis characterizing internal and external early knowledge sharing: the guidance documents and newsletters were crucial for knowledge sharing to be possible, persistence led to increased knowledge sharing over time, and CCNet coaches encompass both direct support and peer encouragement.

The online tracking data revealed considerable *internal* knowledge sharing, but there was relatively little direct evidence of *external* knowledge sharing as we were unable to accurately parse the analytics to separate out internal versus external traffic. However, the survey data indicated that a similar number of individuals shared information internally and externally (although only about half of each group did both), indicating that the low direct evidence of external sharing in the online data is likely due to data limitations. It is unsurprising that newsletters were an effective means to motivate staff to learn more about CbD 2.0 (the consideration phase of diffusion), as they made it easy (via direct links) and had a very small time-cost to read. While emails sent out by several executives were collectively only opened by roughly half of the staff who received them, we believe image-blocking software contributed to undercounting email opens (revealing the limitations of using this type of data, as tracking only works when images are loaded and some staff have images blocked by default). If email content was embedded in images and the email text made that clear, it is likely more staff would have loaded the images allowing the tracking to function. Finally, it was not surprising that in general people were more likely to seek knowledge about CbD 2.0 if it was either more relevant to their job (e.g. scientists and executives), or if they had been targeted for communications (staff in the North America region).

We cannot definitively omit the possibility that we did not detect external diffusion activity because it did not actually occur, but our approach takes considerable steps to eliminate alternative explanations (and the survey data gives us confidence that it happened). If we had hosted redundant copies of the key documents on both the intranet and public sites, it may have helped to make this distinction, but also could have hindered knowledge sharing as staff would have to find a different link before sharing.

Some of the significant characteristics of individuals who shared knowledge internally confirmed our expectations, particularly: being a CCNet coach (meaning that diffusing CbD internally and externally is part of your job), having increased exposure to CbD (whether passive or active, both indicating you were aware of the innovations), and having already adopted practices incorporating people into their work. Similarly, it was unsurprising that external knowledge sharing was more likely from individuals who also shared knowledge internally, had already adopted practices recommended by CbD 2.0 prior to the document being released, or had adopted practices in response to reading CbD 2.0 guidance.

While participating in online trainings of any kind was weakly associated (p<0.1) with internal knowledge sharing (and we did not expect much of an effect), the finding that in-person trainings was negatively associated with external knowledge sharing was counter to our hypothesis. This may indicate that knowledge-seeking rather than the network-building aspect of trainings is most significant in promoting knowledge sharing (or identifying people prone to knowledge sharing). While the finding that the total number of years in conservation (both at TNC and elsewhere) has a very slight negative effect on internal knowledge sharing was unexpected, it is possible that these staff are more entrenched due to their experience, and less interested in learning new ways to practice conservation. Finally, while we anticipated that job grade would not significantly affect diffusion, it is plausible that more senior staff were more aware of the organizational importance of CbD 2.0, leading them to pass it on to their direct reports and colleagues. Unfortunately, we do not have a satisfactory explanation as to why most of the significant characteristics were only significant for either internal or external knowledge sharing (with only prior “people” practices significant to both).

### Opportunities to improve diffusion

Our quantitative findings point to several potential ways to improve knowledge sharing at large organizations, and the interviews provide some insight into adoption as well. Our key recommendations are for organizations to use repeated broadly targeted communications to promote knowledge sharing, to review the factors from the interviews found to aid diffusion overall, use internal data to identify staff playing key roles in diffusion to foster that behavior, and to consider sharing data with academics to encourage diffusion via published literature.

First, while relatively simple and low-cost forms of communications such as newsletter stories and webinars were found to be effective ways to get staff to seek and share knowledge, persistent and repeated communications were key as the cumulative number of staff reached over time steadily increased. Thus, a single announcement will not suffice to catch the attention of a target audience, but that doesn’t mean that more expensive in-person workshops are necessary simply to spread knowledge (although interviews revealed that these workshops are important for *adoption*). Communications are critical given our findings that both simply passively receiving information, and making it easy for individuals to actively seek out more information was associated with greater knowledge sharing. The use of common language and methods around evidence based practices can help to build the legitimacy, shared understanding, and trust needed for sustained collaborations [4].

Second, the factors which were listed as important to promote or hinder diffusion during the interviews should be useful to others seeking to improve diffusion. See the Results section for details, but the point that knowledge sharing alone will not suffice to drive adoption is a key lesson.

Third, organizations can use internal data to identify staff likely to be aiding diffusion. The finding that staff who complete more online trainings were more likely to share knowledge internally (confirming the findings of [26]) indicates that investing in the professional development of staff through relatively low-cost online programs could aid diffusion. However, it is possible that staff who take more trainings are more likely to share information, but not *because* of the trainings (meaning this variable could be an identifier rather than a driver). Organizations could identify staff who are taking advantage of these resources, and target them for further professional development to boost their effectiveness (although there could be privacy concerns with identifying staff in this way). Furthermore, the importance of the CCNet coaches in internal knowledge sharing demonstrates their value to TNC in not only training staff on existing methods, but in adapting as the methods evolve as well.

Finally, the finding that there is a substantial body of literature about CbD published without any TNC authors reveals that organizations may wish to consider collaborating with academics on these kinds of articles (and sharing them) as a potentially useful pathway of diffusion. Many scientists regularly monitor the peer-reviewed literature, and publishing is one way to broaden the audience reached on a given topic.

### How to improve future studies

As noted above, the range of data used in this study to examine diffusion represents a significant advancement in the study of knowledge diffusion at large organizations, and we recommend that future research consider how to incorporate similar breadth and diversity of data. Nonetheless, studying diffusion in a large organization such as TNC (which had 3,925 full time employees as of June 2016) is challenging, especially given privacy concerns which limit our ability to monitor communications. Additional case studies are needed.

While we collected several data sources that collectively provide considerable information about diffusion, there is still some early diffusion that we were *not* able to track. For example, we heard anecdotally (in person or via email) about a number of in-person workshops pertaining to CbD 2.0 that we did not observe in our data. The potentially most important modes of knowledge sharing that we could not observe were conversations that happened via the phone and in person. Additional data could have been captured but was missed due to measurement error or other limits to our methods. For example, one paper mentioned in interviews as critical for external diffusion [38] was originally missed as it did not include the phrase "conservation by design" (instead citing “A Geography of Hope” in the acknowledgments, which was the title of the first version of CbD). Finally, when we surveyed staff to assess which CbD 2.0 practices they already used, we may have lumped together the few people who were already using the practice as recommended and many people who were practicing a limited form of the practice. For example, 419 of 686 respondents to our survey (60.7%) reported that they were already "incorporat[ing] evidence in the conservation planning process" but it is likely that they were not doing so to the degree called for by CbD 2.0.

We also found distinguishing between internal and external knowledge sharing to be challenging using online metrics. While we have several sources of data that were limited to TNC staff, the publicly available resources were also found to be visited frequently by TNC staff. For example, the CbD 2.0 preview was published on nature.org (a public website) in March 2015, but traffic spiked most sharply in October 2015, shortly after TNC staff were sent a link to this preview and invited to review draft guidance. Similarly, the full guidance document was on a public site, and we had no reliable way to split which page views were internal vs. external. This problem was compounded when internal and external outreach efforts happened simultaneously (e.g. the day the full guidance was released, emails went out to several TNC executives with a link to the guidance, but the same link was posted to the external CCNet listserv), making it challenging to interpret spikes in activity.

We only measured whether or not staff shared information about CbD 2.0, and were *not* able to track whether the people they shared with in turn shared it further (e.g., cascading forwards of the information). We are also not able to account for whether or not the people they shared information with were people who need to know about the work or not (e.g. did they share knowledge with “the right people”). Understanding the quantity of quality of knowledge sharing would be good topics for future research to better determine the total scope and impact of the sharing. Questions such as these could have been answered with metadata about (and content of) emails sent by individuals. While there are important and valid privacy concerns, and we would not support giving researchers access to read the email of other staff (or to identify individuals who sent certain emails), there is a lot of room for analysis that robustly protects privacy. For example, all email content could be ignored, but email headers (metadata including only the sender, recipient, and subject) could be analyzed solely to count communications between different staff and build social network data. Another possibility would be to count email chains that match certain keywords (like "conservation by design"), so that again the content would not be visible, but emails that match would be identified. Upon review with TNC's ethics department we decided not to use email data because despite the strong privacy protections we proposed, there was a risk that employees could still perceive a potential violation of privacy.

As noted above, we identify characteristics of staff who share information (both internally and externally), but were unable to identify what happened after they shared that information. Future work should examine the cascading effect of knowledge sharing (which staff are sharing information that continues to spread as others share it, leading to a greater total number of people receiving the information), as well as the quality of knowledge sharing (which staff are sharing the information with people who can directly use the information, and which staff are actually driving adoption of the innovation). This should also include studying later stages of knowledge diffusion (including adoption), which could include broader surveys, reviews of conservation websites to look for specific language, and analysis of discussion boards like CCNet.

Finally, this study investigated and observed the process of the diffusion of an innovation, and what factors seemed to play a significant role in aiding or hindering it, but did not explore the underlying motivations as to why individuals did or did not choose to engage in the various stages of knowledge diffusion. Future research should go beyond observing diffusion and also seek to identify the underlying motives which drive individuals to participate in diffusion (or not).

Our study offers several contributions, built primarily on the large and robust data sources we had access to (see Table 1 for the full list, including the sources we considered but did not use). We quantified the internal spread of knowledge about CbD 2.0 at TNC, and identified the kinds of events driving it. We also showed that external knowledge sharing and dissemination was happening via the published literature to other sectors beyond TNC and other conservation NGOs. Our analysis identified characteristics associated with both internal knowledge sharing (i.e., within TNC) and external diffusion (i.e., outside of TNC) at the individual level. While we are unable to fully track the spread of knowledge via email and word of mouth, we believe the range of methods used together account for a good overview of the early phases of diffusion.

## Supporting information

S1 File Complete list of 151 publications that cite or discuss CbD. This set of publications was winnowed down from an initial set of 295 publications from Google Scholar and Web of Science between 1995–2015.
(XLSX)

S2 File Survey questionnaire. The full set of questions used in the survey of all conservation, science, and executive staff within the North America program of TNC (1,536 staff), conducted via Qualtrics in May 2016.
(PDF)

S1 Table Comparison of sample used for analysis to total number of survey recipients. (DOCX)

S2 Table Detailed description of variables compiled to characterize internal and external knowledge sharing. (DOCX)
