# Supplementary material for: Knowledge diffusion within a large conservation organization and beyond
Source: PLoS One. 2018 Mar 1;13(3):e0193716. doi: 10.1371/journal.pone.0193716 (PMC5832310; doi:10.1371/journal.pone.0193716)
Supplement: S2 File — The full set of questions used in the survey of all conservation, science, and executive staff within the North America program of TNC (1,536 staff), conducted via Qualtrics in May 2016. (PDF) [file pone.0193716.s002.pdf]

Default Question Block

For best results, please use Firefox or Internet Explorer browsers.

Dear Colleagues,

The Nature Conservancy (TNC) respectfully requests your time in responding to the following survey.

This survey is for a project to understand how ideas and practices diffuse within TNC. We are not evaluating the effectiveness of you or any program with which you are affiliated. This work will help TNC better understand how conservation practices are spread and adopted.

We plan to collect the following data as part of this research:

1. This 15-20 minute survey
2. Follow up interviews for a small subset of respondents
3. Some administrative data generated from TNC systems, such as the operating unit and geographical region to which you belong

Completing this survey indicates your consent as a participant in this study. Participating in this study is voluntary, and all data collected will be confidential. All information you provide will be de-identified and it will not be linked back to you. Data may be used for future publications, but because all analyses will use de-identified data and aggregate at the group level, this further ensures that no data can be linked to you.

Please select one of the options below.

- Yes, I voluntarily agree to participate in this research.
- No, I do not choose to participate in this research.

Block A and B1

QUESTIONS A1-A3 ASK ABOUT YOUR BASIC DEMOGRAPHIC INFORMATION AND EDUCATIONAL HISTORY.

A1. Please indicate your gender below.

- Male
- Female

A2. Please indicate your age below.

	Years
Age	

A3. What is the highest level of education you have completed?

- Less than High School
- High School/GED
- Some college
- 2 year College Degree
- 4 year College Degree
- Master's Degree
- Doctoral Degree
- Professional Degree (e.g. JD/MD)

QUESTIONS A4-A5 ASK MORE SPECIFICALLY ABOUT YOUR EDUCATIONAL TRAINING.

A4. Please indicate any formal training you have received and the subject and major of study.
Please select your subject and choose your major from the pull down options below.

Subject
Major

A5. Please indicate any other formal training you have received and the subject and major of study.
Please select your subject and choose your major from the pull down options below.

Subject
Major

QUESTIONS A6-A7 ASK ABOUT YOUR CURRENT POSITION AND YOUR PRIOR PROFESSIONAL EXPERIENCE.

A6. As of January 2015, how many years have you worked in your current position at TNC?

	Years
Years worked in current position	

A7. How many years, including your time within TNC, have you worked in conservation?

	Years
Years worked in conservation	

THE FOLLOWING B1-B2 ITEMS ASK QUESTIONS REGARDING YOUR COLLEGIAL RELATIONSHIPS.
Please note: we will not identify you or share your responses to these survey questions with your colleagues.

B1. Please list *up to fifteen of your closest colleagues* (those people from whom you seek advice or with whom you frequently interact within a professional context) within TNC and the frequency of your interaction. You do not need to fill in all of the blanks.

Names will autofill as you type. Begin, by typing the last name of your colleague, then first name.

	Yearly	Monthly	Weekly	Daily
Full Name of Colleague 1	○	○	○	○
Full Name of Colleague 2	○	○	○	○
Full Name of Colleague 3	○	○	○	○
Full Name of Colleague 4	○	○	○	○
Full Name of Colleague 5	○	○	○	○
Full Name of Colleague 6	○	○	○	○
Full Name of Colleague 7	○	○	○	○
Full Name of Colleague 8	○	○	○	○
Full Name of Colleague 9	○	○	○	○
Full Name of Colleague 10	○	○	○	○
Full Name of Colleague 11	○	○	○	○
Full Name of Colleague 12	○	○	○	○
Full Name of Colleague 13	○	○	○	○
Full Name of Colleague 14	○	○	○	○
Full Name of Colleague 15	○	○	○	○

Block B2

B2. Please identify general areas of communication or collaboration below.
(Please choose all that apply)

	Is this a person with whom you consult or seek advice?	What type of work do you communicate with this person about? *(check all that apply)*				Please indicate if you have done the following with this person: (check all that apply)		
	Yes	Science	Conservation Program Activities	Fundraising or Proposal Development	Managment	Attended Any Training	Shared data, reports or methodology	Partnered or collaborated on a project
${q://QID98/ChoiceTextEntryValue/1}								
${q://QID98/ChoiceTextEntryValue/2}								
${q://QID98/ChoiceTextEntryValue/3}								
${q://QID98/ChoiceTextEntryValue/4}								
${q://QID98/ChoiceTextEntryValue/5}								
${q://QID98/ChoiceTextEntryValue/6}								
${q://QID98/ChoiceTextEntryValue/7}								
${q://QID98/ChoiceTextEntryValue/8}								
${q://QID98/ChoiceTextEntryValue/9}								
${q://QID98/ChoiceTextEntryValue/10}								
${q://QID98/ChoiceTextEntryValue/11}								
${q://QID98/ChoiceTextEntryValue/12}								
${q://QID98/ChoiceTextEntryValue/13}								
${q://QID98/ChoiceTextEntryValue/14}								
${q://QID98/ChoiceTextEntryValue/15}								

Block B3

B3. Please list *up to fifteen of your colleagues* within TNC that have communicated with you about the CbD 2.0 Guidance and/or the Human Well-Being and Conservation Tutorial (HWB Tutorial). You do not need to fill in all of the blanks.

Communications can include email, phone, and in-person conversations. Communications could come from your colleague or from you.

Names will autofill as you type. Begin, by typing the last name of your colleague, then first name.

	CbD 2.0 Guidance		HWB Tutorial	
	Heard about CbD 2.0 Guidance from this person (in-person or virtually)	Told this person about CbD 2.0 Guidance (in-person or virtually)	Heard about the HWB Tutorial from this person (in-person or virtually)	Told this person about the HWB Tutorial (in-person or virtually)
Full Name of Colleague 1	○	○	○	○
Full Name of Colleague 2	○	○	○	○
Full Name of Colleague 3	○	○	○	○
Full Name of Colleague 4	○	○	○	○
Full Name of Colleague 5	○	○	○	○
Full Name of Colleague 6	○	○	○	○
Full Name of Colleague 7	○	○	○	○
Full Name of Colleague 8	○	○	○	○
Full Name of Colleague 9	○	○	○	○
Full Name of Colleague 10	○	○	○	○
Full Name of Colleague 11	○	○	○	○
Full Name of Colleague 12	○	○	○	○
Full Name of Colleague 13	○	○	○	○
Full Name of Colleague 14	○	○	○	○
Full Name of Colleague 15	○	○	○	○

Block C1

C1. Please indicate if in the past 12 months you have engaged in the following activities when *leading, developing, or adaptively managing conservation efforts*.

	Yes
**Analyze and identify new opportunities by conducting:**	
A situation analysis that includes considerations for people and nature	☐
Strategy and opportunity mapping	☐
An ecoregional assessment	☐
Gap analysis	☐
Biodiversity or habitat status, threats, or thresholds assessments	☐
Ecosystem service targets, or threats assessments	☐
Analysis of political, economic, and social factors conducive for conservation work	☐
Analysis of conservation priorities based on multi-objective criteria (e.g., nature and people considerations)	☐
Some type of systematic review or synthesis of the peer reviewed, grey, and white literature	☐
Stakeholder analysis that includes explicit consideration of all relevant groups that will be affected	☐
**Conduct field research or modeling to:**	
Support development/testing of strategies	☐
Identify new conservation targets, threats, and trade-offs	☐
Develop innovative methods	☐

Please indicate the percent of time you spend *leading, developing, or adaptively managing conservation efforts* (i.e., all of the items above) .

	Percentage of time
Engaging in *leading, developing, or adaptively managing conservation efforts*	0

Block C2

C2. Please indicate if in the past 12 months you have engaged in the following activities when *analyzing and building evidence for conservation*.

	Yes
**Identify conservation opportunities by conducting or participating in:**	
Developing a Conservation Action Plan (CAP)	☐
Creating a Conservation Business Plan	☐
Developing theory of change linked and informed by data (e.g., grey, white, peer reviewed literature, key informant interviews, etc.)	☐
Developing a theory of change or results chain articulating how conservation strategies can negatively or positively impact people	☐
**Evaluate alternative strategies for taking advantage of conservation opportunities by:**	
Reading the conservation literature (e.g., government or policy reports, peer reviewed literature)	☐
Consulting with trusted friends and colleagues	☐
Monitoring by field research or modeling	☐
Conducting original research to identify new conservation targets, threats, and trade-offs	☐
Facilitating conversations about people and nature	☐
**Evaluate the effect or impact of conservation outcomes by:**	
Qualitative assessment (e.g., key informant interviews, focus groups, administrative documents or photographs)	☐
Quantitative monitoring to detect trends or impact	☐
Updating strategic plan after acquiring new information	☐
Develop indicators for human well-being outcomes related to a conservation strategy	☐
Analyzing socioeconomic data	☐

Please indicate the percent of time you spend *analyzing and building evidence for conservation* (i.e., all of the items above).

Please note: These percentages do not need to sum to 100%.

	Percentage of time
Engaging in *analyzing and building evidence for conservation*	0

Block C3

C3. Please indicate if in the past 12 months you have engaged in these aspects of *communicating conservation science and activities*.

	Yes
**Share science or technical information:**	
Within TNC	☐
Other conservation entities	☐
Other audiences (e.g. corporations, non-conservation oriented government, academic departments, or NGOs)	☐
**Build capacity with internal and external audiences on methods, tools and/or best practices related to:**	
Conservation priorities and strategies	☐
Human well-being outcomes or impacts of our work	☐
Nature conservation outcomes or impacts of our work	☐
Innovative technical or scientific methods and tools	☐
Equity or the consideration of traditionally disadvantaged stakeholders	☐
**Promote conservation work to:**	
Facilitate conversations about people and nature strategies and outcomes	☐
Incorporate cross- or multi-disciplinary knowledge	☐
Establish new partnerships with outside organizations to address new disciplinary perspectives and expertise	☐
Raise additional funds for existing or new projects	☐
Audiences outside of conservation	☐
Coordinate learning exchanges and conferences	☐

Please indicate the percent of time you spend engaging in practices related to *communication of conservation science and activities* (i.e., all of the items above)

Please note: These percentages do not need to sum to 100%.

	Percentage of time
Engaging in practices related to *communication of conservation activities*	0

Block C4

C4. Please indicate if in the past 12 months, you have engaged in these aspects of *conservation management*.

	Yes
**Developed and led:**	
Conservation visioning exercises	☐
Efforts to build an evidence base to inform strategies and priorities	☐
Effort to consider how the Human Subjects Research SOP applies to your work and what, if any, actions need to be taken	☐
**Cultivated:**	
Partnerships with other conservation or natural resource management organizations and government agencies	☐
Partnerships with non-conservation oriented organizations, such as corporations, governments or academic departments, or other NGOs	☐
Non-conservation oriented audiences for fundraising	☐
Fundraising support to build an evidence base	☐

Please indicate the percent of time you spend engaging in practices related to *conservation management* (i.e., all of the items above)

Please note: These percentages do not need to sum to 100%.

	Percentage of time
Engaging in practices related to *conservation management*	0

Block D

THE FOLLOWING ITEM, D1, ASKS ABOUT YOUR PERCEPTIONS AND WORK RELATED PRACTICES.

D1. I use the following sources to acquire information on conservation practice and science to share with others:

	Never or almost never	1-3 times per year	1-3 times per month	1-3 times per week
Peer reviewed scientific literature	○	○	○	○
Reports, white papers, grey literature	○	○	○	○
Popular articles, news stories, blogs, and other news sources	○	○	○	○
Internal conferences, trainings (e.g., from Learn@TNC), or other training workshops	○	○	○	○
CONNECT or ReCONNECT Staff News Emails	○	○	○	○
Discussions with colleagues within TNC	○	○	○	○
Discussions with colleagues outside TNC	○	○	○	○
Electronic newsletters and listserv	○	○	○	○
Print outreach and communication materials	○	○	○	○
Other non-profit nature organizations	○	○	○	○
Other (if so, please specify below) ______	○	○	○	○

Block E

QUESTION E1 EXPLORES WHAT CONSERVATION MEANS TO YOU.

E1. These questions ask about your thoughts on *conservation organizations,* which are organizations that protect, preserve, or restore nature. Please indicate your extent of agreement to the following statements.

	Strongly Disagree	Disagree	Agree	Strongly Agree	Don't Know
Conservation organizations can protect the environment and improve human well-being	○	○	○	○	○
Projects to increase biodiversity should only be done when they increase human well-being.	○	○	○	○	○
The environment should be protected even if it decreases human well-being.	○	○	○	○	○
The environment can be protected without affecting human well-being	○	○	○	○	○
Protecting the environment should only be done when it also increases human well-being.	○	○	○	○	○
Conservation organizations should only protect the environment when it improves human well-being.	○	○	○	○	○
Projects to protect the environment should do so even at the cost of human well-being.	○	○	○	○	○

Block F

QUESTIONS F1 AND F2 EXPLORE WHAT AREAS YOUR WORK INVOLVE.

F1. What Whole System and North American Regional (NAR) Priorities does your work involve?

Please select all the Whole Systems and NAR Priorities you are involved in. If you are not involved in any Whole System and NAR priority, please select *not applicable*

	My work involves this Whole System or NAR Priority
Not applicable (N/A)	☐
Advance integrated ocean and coastal management (IOCM)	☐
Atlantic Sound	☐
Canadian Boreal Forest	☐
Caribbean Coasts and Marine Protected Areas	☐
Central Appalachian Mountains	☐
Central Great Plains Grasslands	☐
Chesapeake Bay	☐
Colorado River Basin	☐
Connecticut River	☐
Crown of the Continent	☐
Emerald Edge	☐
Everglades	☐
Great Lakes	☐
Gulf of Mexico	☐
Hawaiian Island Ecosystems	☐
Longleaf Pine Forests	☐
Mississippi River	☐
Mojave Desert Ecoregion	☐
Promote integrated water resource management	☐
Puget Sound	☐
Restore America's forests	☐
Restore coastal fisheries for people and nature	☐
Secure benefits to nature from energy development	☐
Urban conservation in North America cities	☐

F2. What Global Challenges Global Solutions (GCGS) areas and/or North American Regional project does your work involve?

Please select all Global Challenges Global Solutions (GCGS) and/or North American Regional project you are involved in. If you are not involved in any GCGS or North American Regional project, please select *not applicable.*

	My work involves this GCGS or North American Regional project
Not applicable (N/A)	☐
Climate and Energy	☐
Climate Risk & Resilience	☐
Indigenous & Communal Conservation	☐
Development by Design	☐
Forests and Climate	☐
Global Fisheries	☐
Great Rivers	☐
Ocean Solutions	☐
Securing Water	☐
Global Agriculture	☐
Resilient Cities	☐

Block G

QUESTIONS G1-G8 ASK ABOUT CbD 2.0. THIS INCLUDES THE OVERVIEW DOCUMENT RELEASED IN MARCH 2015, AS WELL AS THE GUIDANCE DOCUMENT RELEASED IN MARCH 2016.

G1. Please tell us if you do the following as a result of CbD 2.0

	Yes	No	Don't Know	Already practiced before CbD 2.0
Incorporate evidence in the conservation planning process	○	○	○	○
Incorporate uncertainty in the conservation planning process	○	○	○	○
Use Human Subjects SOP	○	○	○	○
Measure our impacts on people	○	○	○	○
Use standards of measurement (e.g., SMART) for monitoring and evaluating our impacts on people	○	○	○	○

G2. How much have you engaged in the following practices since CbD 2.0 was introduced?

	More	Less	The same
Incorporate evidence in the conservation planning process	○	○	○
Incorporate uncertainty in the conservation planning process	○	○	○
Use Human Subjects SOP	○	○	○
Measure our impacts on people	○	○	○
Use standards of measurement (e.g., SMART) for monitoring and evaluating our impacts on people	○	○	○

G3. How has your OU diversified the number of funding sources in the past 12 months?

- ○ More
- ○ Less
- ○ The same
- ○ Don't know

G4. How many external partners (e.g., agencies or organizations) have you worked with in the past 12 months?

G5. How much have you shared CbD 2.0 knowledge or guidance with outside collaborators?

	None (0)	A Little (1-3 people)	Some (4-6 people)	A Lot (7+ people)
Degree of sharing	○	○	○	○

G6. These questions ask about how CbD 2.0 influenced your work. Please indicate the extent you agree with the following statements.

	Strongly Disagree	Disagree	Agree	Strongly Agree
Your outside collaborators engage in practices consistent with CbD 2.0	○	○	○	○
Applying the CbD 2.0 approach has increased the types of activities we do that can be funded	○	○	○	○
Applying the CbD 2.0 approach has increased the number of partners we can work with outside of TNC	○	○	○	○
Applying the CbD 2.0 approach has increased the number of constituents we serve	○	○	○	○
Applying the CbD 2.0 approach has increased the expertise we have access to	○	○	○	○
Applying the CbD 2.0 approach has increased our funding sources.	○	○	○	○
Applying the CbD 2.0 approach has increased the number of contexts in which we can work	○	○	○	○

G7. Have you downloaded the CbD 2.0 Guidance document, which was released on March 2016?

- ○ Yes
- ○ No

G8. Have you read all or part of the CbD 2.0 Guidance document, which was released on March 2016?

- ○ Read all
- ○ Read some
- ○ Did not read

Browser Meta Info

This question will not be displayed to the recipient.
Browser: **Firefox**
Version: **46.0**
Operating System: **Macintosh**
Screen Resolution: **1440x900**
Flash Version: **21.0.0**
Java Support: **1**
User Agent: **Mozilla/5.0 (Macintosh; Intel Mac OS X 10.11; rv:46.0) Gecko/20100101 Firefox/46.0**
