# Supplementary material for: Knowledge diffusion within a large conservation organization and beyond
Source: PLoS One. 2018 Mar 1;13(3):e0193716. doi: 10.1371/journal.pone.0193716 (PMC5832310; doi:10.1371/journal.pone.0193716)
Supplement: S1 Table — (DOCX) [file pone.0193716.s003.docx]

S1 Table. Comparison of Sample Used for Analysis to Total Number of Survey Recipients.

|  | **Population (N=1536)** | **Sample(N=686)** |
| --- | --- | --- |
| Service years | *Two sided t-test*  t = -1.4852, df = 1351.6, p-value = 0.1377 | |
| Job grade | *One sided t-test with alternative=”greater”*  t = -5.3066, df = 1471.1, p-value = 0.9899 | |
| Operating Unit frequency | *Regional/State*=0.082 | *Regional/State*=0.111 |
| Job family frequency | *Conservation/Executive*=13.76  *Conservation/Science*=5.77  *Executive/Science*=0.42 | *Conservation/Executive*=8.73  *Conservation/Science*=4.67  *Executive/Science*=0.53 |
