# Supplementary material for: Knowledge diffusion within a large conservation organization and beyond
Source: PLoS One. 2018 Mar 1;13(3):e0193716. doi: 10.1371/journal.pone.0193716 (PMC5832310; doi:10.1371/journal.pone.0193716)
Supplement: S2 Table — (DOCX) [file pone.0193716.s004.docx]

S2 Table. Detailed Description of Variables Compiled to Characterize Internal and External Knowledge Sharing. This table provides additional detail about the variables from Table 2.

| **Variable Name** | **Description** | **Data Source** | **Data Preparation** |
| --- | --- | --- | --- |
| ***Dependent variables*** | | | |
| Individual shared info internally | Individual shared information with other TNC staff | 2016 Survey | Q B.3 Binary response sourced directly |
| Individual shared info externally | Individual shared information externally | 2016 Survey | Q G.5 Degree of sharing information collapsed to binary type such that “shared to none”=0 and “shared to 1-3 people”, “shared to 4-6 people”, “shared to 7+ people) =1 |
| ***Independent variables*** | | | |
| *Sharing information* | | | |
| Sharing science/tech information internally | Shared with other TNC staff | 2016 Survey | Q C.3 Binary responses sourced directly |
| Sharing science/tech information externally | Shared with other conservation entities or other audiences (e.g., corporations, NGOs) | 2016 Survey | Q C.3 Binary responses sourced directly |
| *Formal role/job in knowledge sharing* | | | |
| CCNet Coach | Individual is a CCNet Coach | CCNet Subscriber List | Directly sourced |
| *Awareness, knowledge and exposure* | | | |
| # of online trainings completed | Completed optional professional development trainings hosted online for TNC staff | TNC Training / Learning | Sum of the online trainings for each individual |
| # of in person trainings completed | Completed optional professional development “classroom” trainings hosted for TNC staff | TNC Training / Learning | Sum of the in person trainings for each individual |
| CbD awareness (passive) | Indicators that the individual passively received information about CbD 2.0 (e.g. receiving an email they may or may not have read or acted on), through one of 7 indicators we have data for. | Assorted | Sum of the 7 passive indicators, where 1 indicates the individual received information passively via that pathway. |
| CbD knowledge seeking (active) | Indicators that the individual actively sought out information about CbD 2.0, e.g. visiting a website or reviewing the guidance document | Assorted | Sum of the 20 possible active knowledge seeking indicators, where 1 indicates the individual engaged in that active behavior. |
| *Network characteristics* | | | |
| Boundary spanner status |  | Derived from TNC Labor data | Boundary spanners identified via social network mapping based on Labor data, see Reddy et al (in preparation for details) |
| Years working in conservation | # of years working in the conservation field | 2016 Survey | Q A.7 Directly sourced |
| # of internal collaborators | # of TNC staff which an individual attended a training, shared data or methodologies, or partnered with on a project | 2016 Survey | Q B.2 Sum of the internal collaborators |
| Service years at TNC | # of years employed at TNC | TNC Admin Data | Directly sourced |
| *Changes in practice post CbD 2.0* | | | |
| Incorporates evidence due to CbD 2.0 | Incorporating evidence in the conservation planning process in response to reading CbD 2.0 | 2016 Survey | Q G.1 Binary response such that “Yes”=1, “No”, “Don’t know” & “Already practiced”=0 |
| Incorporates uncertainty due to CbD 2.0 | Incorporating uncertainty in the conservation planning process in response to reading CbD 2.0 | 2016 Survey | Q G.2 Binary response such that “Yes”=1, “No”, “Don’t know” & “Already practiced”=0 |
| *Existing alignment/identification with innovation* | | | |
| Prior “people” practices | Responses related to individual already incorporating people in conservation prior to CbD 2.0 | 2016 Survey | Sum of the 8 binary “people in conservation” responses |
| Prior “evidence” practices | Responses to individual already practicing evidence based conservation prior to CbD 2.0 | 2016 Survey | Sum of the 11 binary “evidence based” responses |
| Prior “Systematic change” practices | Responses to individual already considering systematic change prior to CbD 2.0 | 2016 Survey | Sum of the 3 binary “systematic change” responses |
| *Communication* | | | |
| Percent of time spent in communication | Percentage of total time spent communicating about conservation activities | 2016 Survey | Q C.3 Directly sourced |
| # of staff communicated with | # of staff recipient communicated with about science and/or conservation activities | 2016 Survey | Q B.2 Sum of the # of staff that the recipient indicated they communicate with about science and/or conservation |
| *Additional variables* | | | |
| Gender | Male or Female | TNC Admin Data | Directly Sourced |
| Job Grade (higher is more senior) | Range is 1 to 10, with higher grade meaning a more senior position | TNC Admin Data | Directly Sourced |
| Years post-secondary education | Range is 2 to 8, representing the number of years spent in post-secondary education (up to a maximum of 8) | TNC Admin Data | Directly Sourced |
| Operating Unit (OU) Size | # of people in an Operating Unit | TNC Admin Data | Directly sourced |
| Budget/person | Total budget allocated to each staff person in their Operating Unit | TNC Admin Data | Total budget per OU/ OU Size |
| OU Type | Type of Operating Unit (state / regional / central) | TNC Admin Data | Directly sourced |
